# Association Between Blood Pressure Variability and Short-Term Outcome After Intra-arterial Thrombectomy in Acute Stroke Patients With Large-Vessel Occlusion

**DOI:** 10.3389/fneur.2020.604437

**Published:** 2021-01-11

**Authors:** Mengqi Yang, Tao Lu, Baohui Weng, Yi He, Hong Yang

**Affiliations:** ^1^Neurology and Stroke Center, The Fourth Affiliated Hospital of Guangxi Medical University, Liuzhou, China; ^2^Medical Records Room, The Fourth Affiliated Hospital of Guangxi Medical University, Liuzhou, China

**Keywords:** blood pressure variability, large-vessel occlusion, stroke, intra-arterial thrombectomy, outcome

## Abstract

The optimal range of blood pressure variability (BPV) for acute stroke patients with large-vessel occlusion (LVO) remains unclear. This study investigated the association between BPV from admission through the first 24 h after intra-arterial thrombectomy (IAT) and short-term outcome in LVO patients. We retrospectively analyzed 257 consecutive patients with LVO stroke who were treated with IAT. BP values were recorded at 2-h intervals from admission through the first 24 h after IAT. BPV, as reflected by pulse pressure variability (PPV), was determined based on standard deviation (SD), coefficient of variation (CV), successive variation (SV), and the difference between maximum and minimum blood pressure (ΔBP; systolic BP minus diastolic BP). The association between BPV and clinical outcome (Modified Rankin Scale score at 90 days) was analyzed by multivariate logistic regression analysis. Of the 257 included patients, 70 had a good outcome at 3 months. PPV from admission through the first 24 h after IAT was independently associated in a graded manner with poor outcome [multivariable-adjusted odds ratios (95% confidence interval) for the highest of PPV were 43.0 (8.7–212.8) for SD, 40.3 (9.8–165.0) for CV, 55.0 (11.2–271.2) for SV, and 40.1 (8.0–201.9) for ΔBP]. The area under the receiver operating characteristic curve (95% confidence interval) of the PPV parameters were 0.924 (0.882–0.965) for SD, 0.886 (0.835–0.938) for CV, 0.932 (0.891–0.973) for SV, and 0.892 (0.845–0.939) for ΔBP, and the Youden index values were 0.740, 0.633, 0.759, and 0.756, respectively. In summary, BPV from admission through the first 24 h after IAT was independently associated with poor outcome at 3 months in patients with LVO, with greater variability corresponding to a stronger association. Thus, PPV may be a clinically useful predictor of functional prognosis in LVO patients treated with IAT.

## Introduction

Early endovascular treatment (EVT) is a treatment option for acute ischemic stroke (AIS) patients with large-vessel occlusion (LVO) (1, 2). Blood pressure (BP) management during the perioperative period of EVT is important because it is associated with clinical outcome (3). BP variability (BPV) is the fluctuation of BP over a given period of time. Recent studies have demonstrated that BPV is associated with poor functional outcome in AIS patients who have undergone EVT (4–7). However, there is no standard approach for BP management in the acute phase of ischemic stroke (8, 9), and the relationship between BPV and the clinical outcome of AIS patients who have undergone EVT is unclear. Pulse pressure (PP) more accurately reflects BP dynamics than standard BP parameters [e.g., systolic (S)BP or mean arterial pressure] and was shown to be a stronger predictor of stroke and other major vascular events (10). Accordingly, PP variability (PPV)—which is a clinically useful parameter for monitoring intracranial hemodynamics in AIS (11)—may better represent BPV than SBP because it encompasses the pulsatile component of BP (12). However, previous studies have calculated the BPV of SBP and diastolic (D)BP separately, and there have been few reports on the degree of PPV in the acute phase of AIS patients who have undergone intra-arterial thrombectomy (IAT) and its relationship with clinical outcome. This was addressed in the present study by categorizing PPV parameters into tertiles and assessing the strength of the relationship between PPV and the short-term clinical outcome of AIS patients treated with IAT.

## Materials and Methods

### Patients

We retrospectively identified consecutive AIS patients with LVO who underwent emergency IAT at the stroke center of The Fourth Affiliated Hospital of Guangxi Medical University from October 2015 to January 2020. Patients who met the following criteria were enrolled: (1) age ≥18 years; (2) AIS identified by head computed tomography (CT) or magnetic resonance imaging (MRI) at admission and LVO diagnosed by digital subtraction angiography (DSA); (3) patients underwent IAT within 24 h after symptom onset; (4) prestroke Modified Rankin Scale (mRS) score <2; (5) BP values recorded at 2-h intervals from the time of admission through the first 24 h after IAT; (6) patients had a head MRI or CT scan 24 h after IAT to monitor for hemorrhagic complications; (7) 3-month clinical outcome was estimated based on information obtained by phone or in a face-to-face meeting; and (8) patients with complete medical data. The exclusion criteria were as follows: (1) patients who died or underwent hemicraniectomy within 24 h after IAT; (2) patients who previously underwent open surgery or IAT; (3) patients with coexisting severe systemic diseases, such as heart failure, respiratory failure, renal failure, digestive tract hemorrhage, coagulopathy, and malignancies; (4) patients who were lost to follow-up; and (5) patients with missing data. The management of AIS patients with LVO was adapted from the 2015 (13) and 2018 (14) Chinese guidelines for EVT of AIS. The study protocol was approved by The Fourth Affiliated Hospital of Guangxi Medical University (no. LW2020009). Informed consent was obtained from patients or their guardians.

### Collection of Clinical Data

General demographic data including age, sex, and vascular risk factors, including hypertension, diabetes, hyperlipidemia, coronary artery disease, valvular heart disease, atrial fibrillation, smoking, drinking, and body mass index were collected. Neurologic state at admission was evaluated according to the Trial of ORG 10172 in Acute Stroke Treatment (TOAST), National Institutes of Health Stroke Scale (NIHSS), preoperative Alberta Stroke Program Early CT Score (ASPECTS), and Glasgow Coma Scale (GCS). BP was measured at 2-h intervals from admission through the first 24 h after IAT. We also recorded the responsible vessel, cerebral infarction volume, laboratory data, time from symptom onset to recanalization, degree of recanalization, treatment with intravenous thrombolysis (IVT), and whether intravenous antihypertensive or dehydrating drugs were used. Recanalization was graded based on Modified Thrombolysis in Cerebral Ischemia (mTICI) score, with successful recanalization defined as mTICI grade ≥2b (15). Symptomatic intracerebral hemorrhage (sICH) was diagnosed based on the European Cooperative Acute Stroke Study criterion (16) of any intraparenchymal, intraventricular, or subarachnoid hemorrhage in the post-procedural CT scan associated with a ≥4-point increase in NIHSS score. The clinical outcome was assessed at 3 months using the mRS score.

### BPV Parameters

BP values were obtained at 2-h intervals from admission through the first 24 h after IAT using an automated electronic device on the nonparetic arm of the supine patient. Four BPV parameters were calculated using PP (SBP minus DBP): standard deviation ($SD:\sqrt{\left(1/\left(n-1\right)\overset{n-1}{\underset{i=1}{\sum }}{\left(BP_{i}-BP_{mean}\right)}^{2}\right)}$), coefficient of variation (CV: SD/BPmean×100%), successive variation ($SV:\sqrt{\left(1/\left(n-1\right)\overset{n-1}{\underset{i=1}{\sum }}{\left(BP_{i+1}-BP_{i}\right)}^{2}\right)}$), and the difference between the maximum and minimum BP (ΔBP) (17).

### Clinical Outcome

We used baseline severity adjustment analysis for clinical outcome evaluation (18). Patients were then divided into favorable and unfavorable groups based on the clinical outcome score. Favorable outcome was classified as a 3-month mRS score of 0–1, 0–2, and 0–3 for pretreatment NIHSS score ≤7, between 8 and 14, and >14, respectively.

### Statistical Analysis

Statistical analyses were performed using SPSS v22.0 software (SPSS Inc., Chicago, IL, USA). Continuous variables are described as mean ± SD in the case of normal distribution, and median (interquartile range) for non-normally distributed data. Categoric variables are described as number (percent) of subjects. Quantitative data were analyzed with the independent sample *t*-test or Mann Whitney U test depending on the value properties. Qualitative data were analyzed with the chi-squared test. BPV parameters were divided into tertiles, and the chi-squared test was first used to analyze the linear trend. The association between BPV parameters and poor outcome was determined with a logistic regression model after adjusting for baseline characteristics. The odds ratio and corresponding 95% confidence interval (CI) were obtained to evaluate any associations. The threshold for statistical significance was set at *P* < 0.05 in all tests. The ability of BPV parameters with significant differences in the multivariate logistic regression model to predict outcome was evaluated by receiver operating characteristic (ROC) curve analysis.

## Results

### Clinical Characteristics of the Study Population

A total of 257 AIS patients with LVO met the inclusion criteria, including 163 (63.42%) males. Based on severity-adjusted dichotomization of outcome, 187 patients (72.76%) had poor outcome at 3 months. The baseline clinical characteristics and outcomes of the patients are presented in Table 1. Patients with poor outcomes were older; more likely to have a history of hypertension, coronary artery disease, and valvular heart disease; and had higher blood glucose and plasma d-dimer and lower albumin levels at admission. These patients also had larger cerebral infarction volume and were more frequently treated by intravenous thrombolysis, with greater stroke severity at admission as determined by NIHSS, GCS, and ASPECTS scores. Additionally, more patients in the poor outcome group were treated with intravenous antihypertensive and dehydrating drugs.

**Table 1:** Baseline Characteristics according to 3-month outcome.

**Variable**	**Favorable outcome (*n* = 70)**	**Poor outcome (*n* = 187)**	***P*-value**
Age, years (mean ± SD)	60.06 ± 11.23	64.16 ± 14.78	0.036^*^
Sex, males (*n*, %)	46 (65.71)	117 (62.57)	0.64^†^
**Risk factor**			
Hypertension (*n*, %)	36 (51.43)	121(64.71)	0.052^†^
Diabetes (*n*, %)	14 (20.00)	60 (32.09)	0.057^†^
Hyperlipidemia (*n*, %)	15 (21.43)	33 (17.65)	0.489^†^
Coronary artery disease (*n*, %)	5 (7.14)	37 (19.79)	0.015^†^
Atrial fibrillation (*n*, %)	20 (28.57)	60 (32.09)	0.588^†^
Valvular heart disease (*n*, %)	11 (15.71)	12 (6.42)	0.020^†^
Prior stroke (*n*, %)	7 (10.00)	20 (10.70)	0.871^†^
Drinking (*n*, %)	10 (14.29)	22 (11.76)	0.586^†^
Smoking (*n*, %)	20 (28.57)	53 (28.34)	0.971^†^
BMI, kg/m^2^ (mean ± SD)	23.05± 3.02	23.73 ± 3.46	0.145^*^
**TOAST type** ***n*** **(%)**			
Large artery atherosclerosis *n* (%)	49 (70.00)	125 (66.84)	0.630^†^
Cardioembolism *n* (%)	19 (27.14)	51 (27.27)	0.586^†^
Other reason *n* (%)	0 (0.0)	3 (1.60)	0.565^†^
**Location of occlusion (*****n*****, %)**			
ICA	18 (25.71)	49 (26.2)	0.937^†^
M1	35 (50.0)	77 (41.18)	0.204^†^
M2	6 (8.57)	20 (10.7)	0.615^†^
ACA	1 (1.43)	4 (2.14)	0.999^†^
BA	13 (18.57)	31 (16.58)	0.706^†^
VA	2 (2.86)	11 (5.88)	0.506^†^
Cerebral infarction volume, ml (median, IQR)	16.6 (4.4–47.4)	46.0 (19.0–146.0)	<0.001^‡^
Admission NIHSS (median, IQR)	13 (7–18)	16 (12–19)	<0.001^‡^
Admission GCS (median, IQR)	15 (15–15)	15 (11–15)	0.006^‡^
ASPECTS (median, IQR)	8 (7–8)	7 (6–8)	<0.001^‡^
Symptom onset to recanalization, min (median, IQR)	312 (185–465)	346 (226–526)	0.218^‡^
Successful recanalization (*n*, %)	66 (94.29)	145 (77.54)	0.002^†^
sICH (*n*, %)	2 (2.86)	26 (13.9)	0.011^†^
Intravenous thrombolysis (*n*, %)	15 (21.43)	21 (11.23)	0.036^†^
Antihypertensive (*n*, %)	8 (11.43)	48 (25.67)	0.014^†^
Dehydrating (*n*, %)	11 (15.71)	113 (60.43)	<0.001^†^
**Laboratory variables**			
Hemoglobin, mg/dl (mean ± SD)	130.19 ± 15.69	128.66 ± 19.99	0.565^*^
Albumin, g/L (mean ± SD)	40.01 ± 4.27	38.44 ± 4.69	0.015^*^
Creatinine, mg/dl (mean ± SD)	85.61 ± 32.54	85.88 ± 27.31	0.947^*^
Glucose, mmol/L (mean ± SD)	6.52 ± 2.79	7.75 ± 3.35	0.003^*^
HbA1c, mmol/L (mean ± SD)	5.93 ± 1.60	6.27 ± 1.80	0.162^*^
Hcy, umol/L (mean ± SD)	11.47 ± 4.33	13.05 ± 7.34	0.091^*^
D-dimer, mg/L (median, IQR)	0.96 (0.7–2.7)	1.9 (0.8–4.7)	0.010^‡^

*Good outcome was defined as a 3-month mRS score of 0 to 1 for pretreatment NIHSS scores of was ≤ 7, mRS score of 0–2 for pretreatment NIHSS scores of was 8–14, and mRS score of 0–3 for pretreatment NIHSS scores of was >14*.

*SD, standard deviation; BMI, body mass index; TOAST, the Trial of ORG 10172 in acute stroke treatment; ICA, internal carotid artery; ACA, anterior cerebral artery; BA, basilar artery; VA, vertebral artery; IQR, interquartile range; NIHSS, National Institute of Health Stroke Scale; GCS, Glasgow Coma Scale; ASPECTS, Alberta Stroke Program Early CT Score; sICH, symptomatic intracerebral hemorrhage; HbA1c, hemoglobin A1c; Hcy, homocysteine*.

* *Independent-sample t-tests*.

† *Chi-square tests*.

‡ *Mann Whitney U test*.

### Relationship Between BPV Parameters and Clinical Outcome

The relationship between PPV and a 3-month outcome were analyzed in the successful and unsuccessful recanalization groups with the independent samples *t*-test (Table 2). Maximum and mean PP and all PPV parameters (SD, CV, ΔBP, and SV) of the successful recanalization group were significantly higher in patients with poor outcome. The same was observed in the unsuccessful recanalization group, with the exception of maximum and mean PP and ΔBP. However, only the SD of PPV showed a statistically significant difference (*P* < 0.05) between non-symptomatic and sICH groups, with a higher SD in the latter (Table 3).

**Table 2:** Blood pressure variability parameters between favorable and poor outcomes.

**PP parameter**	**Recanalization**		*P*-value^*^	**Non-recanalization**		*P*-value^*^
	**Favorable outcome(*n* = 4)**	**Poor outcome (*n* = 42)**		**Favorable outcome (*n* = 66)**	**Poor outcome (*n* = 145)**	
PP mean	52.16 ± 7.46	56.59 ± 7.72	<0.001	53.21 ± 2.59	57.78 ± 8.04	0.268
PP max	74.08 ± 11.59	84.97 ± 12.65	<0.001	67.50 ± 16.78	65.86 ± 10.21	0.772
PP SD	8.02 ± 3.86	15.21 ± 4.54	<0.001	9.58 ± 2.55	16.02 ± 3.60	0.001
PP CV	0.16 ± 0.10	0.27 ± 0.09	<0.001	0.18 ± 0.05	0.28 ± 0.06	0.002
PP SV	10.46 ± 5.83	22.32 ± 6.37	<0.001	14.21 ± 4.02	23.12 ± 5.25	0.002
PP ΔBP	38.36 ± 18.90	52.41 ± 16.69	<0.001	32.50 ± 12.79	27.62 ± 16.94	0.579

*PP, Pulse pressure; max, maximum; SD, standard deviation; CV, coefficient of variation; SV, successive variation; ΔBP, maximum–minimum difference*.

*Values are mean ± SD*.

* *Independent-sample t-tests*.

**Table 3:** Blood pressure variability parameters between sICH and non- sICH.

**PP parameter**	**sICH (n=28)**	**non- sICH (n=229)**	P value^*^
PP mean	56.03 ± 6.72	55.54 ± 8.05	0.755
PP max	81.93 ± 10.04	78.39 ± 14.57	0.213
PP SD	15.30 ± 5.62	13.17 ± 5.24	0.045
PP CV	0.28 ± 0.11	0.24 ± 0.10	0.064
PP SV	21.94 ± 7.35	18.96 ± 8.05	0.063
PP ΔBP	49.75 ± 20.43	43.79 ± 18.36	0.111

*PP, Pulse pressure; sICH, symptomatic intracerebral hemorrhage; non-sICH, non-symptomatic intracerebral hemorrhage; max, maximum; SD, standard deviation; CV, coefficient of variation; SV, successive variation; ΔBP, maximum–minimum difference*.

*Values are mean±SD*.

* *Independent-sample t-tests*.

**Figure 1:**
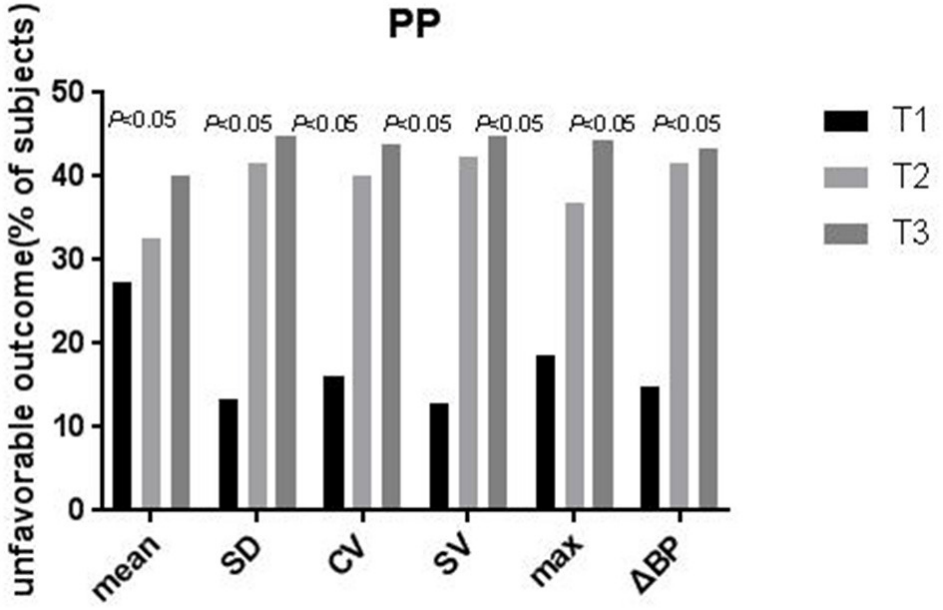
Proportion of patients with unfavorable outcomes at 3-months according to tertiles of the pulse pressure variability parameters. The proportion of patients with unfavorable outcomes Increased significantly across the tertiles of SD, CV, SV, ΔBP, max, and mean (*P*-values are for the X2 test for a linear trend, with the lowest tertile as the reference). PP, pulse pressure; SD, standard deviation; CV, coefficient of variation; SV, successive variation; ΔBP, maximum–minimum difference, max maximum, mean average.

After categorization into tertiles, all PPV variables were associated in a graded manner with poor outcome, as shown by the results of the chi-squared test for linear trends (all *P* < 0.05; Figure 1). In the multivariate logistic regression analysis, all PPV parameters were independently associated in a graded manner with poor outcome, after adjusting for potential confounders including mean PP. The odds of poor outcome were significantly increased in the highest tertile of PPV compared to the lowest tertile (Table 4).

**Table 4:** OR for poor outcome at at 3-months according to the tertile of PP variability indices.

**PP parameter (mmHg)**	**Model 1**		**Model 2**		**Model 3**	
	**OR (95% CI)**	***P*-value^§^**	**OR (95% CI)**	***P*-value^§^**	**OR(95% CI)**	***P*-value^§^**
**PP mean**						
T1 (<52.23)	–	–	–	–	–	–
T2 (52.23–57.87)	2.5 (1.2–5.6)	0.021	2.7 (1.2–6.1)	0.020	–	–
T3 (>57.87)	6.8 (2.7–17.2)	<0.001	6.2 (2.3–17.0)	<0.001	–	–
**PP max**						
T1 (<72.0)	–	–	–	–	–	–
T2 (72.0–84.7)	3.2 (1.5–6.9)	0.004	3.3 (1.5–7.4)	0.004	3.6 (1.4–8.9)	0.006
T3 (>84.7)	45.2(10.4–197.3)	<0.001	44.5 (9.7–204.3)	<0.001	55.0(11.2–271.2)	<0.001
**PP ΔBP**						
T1 (<37.0)	–	–	–	–	–	–
T2 (37.0–49.0)	7.5 (3.3–17.3)	<0.001	7.6 (3.2–17.9)	<0.001	7.0 (2.9–16.7)	<0.001
T3 (>49.0)	56.2(11.4–277.3)	<0.001	51.8 (10.4–258.0)	<0.001	40.1(8.0–201.9)	<0.001
**PP SD**						
T1 (<11.1)	–	–	–	–	–	–
T2 (11.1–15.5)	12.1 (4.8–30.5)	<0.001	12.9 (4.9–36.6)	<0.001	12.8 (4.9–34.0)	<0.001
T3 (>15.5)	55.7 (12.0–258.5)	<0.001	57.9 (11.9–280.9)	<0.001	43.0 (8.7–212.8)	<0.001
**PP CV**						
T1 (<0.20)	–	–	–	–	–	–
T2 (0.20–0.28)	8.6 (3.7–20.1)	<0.001	7.8 (3.3–18.6)	<0.001	9.3 (3.6–23.9)	<0.001
T3 (>0.28)	18.9 (59.0–60.4)	<0.001	21.4 (6.3–72.7)	<0.001	40.3 (9.8–165.0)	<0.001
**PP SV**						
T1 (<16.2)	–	–	–	–	–	–
T2 (16.2–22.5)	20.0 (7.5–20.8)	<0.001	20.4 (7.5–55.6)	<0.001	18.2 (6.6–50.0)	<0.001
T3 (>22.5)	65.9(13.8–314.8)	<0.001	68.9 (14.0–339.0)	<0.001	55.0 (11.2–271.2)	<0.001

*PP, pulse pressure; OR, odds ratio; T, tertile categorization; CI, confidence interval; SD, standard deviation; CV, coefficient of variation; SV, successive variation; ΔBP, maximum–minimum difference, max maximum, mean average*.

*Model 1 was adjusted for cerebral infarction volume, ASPECTS, successful recanalization, intravenous thrombolysis, antihypertensive. Model 2 was adjusted for variables in model 1 variables plus age, hypertension, diabetes, and albumin. Model 3 was adjusted for model 2 variables plus mean PP*.

*The lowest tertile (T1) is reference*.

§ *Multivariable logistic regression analysis*.

**Figure 2:**
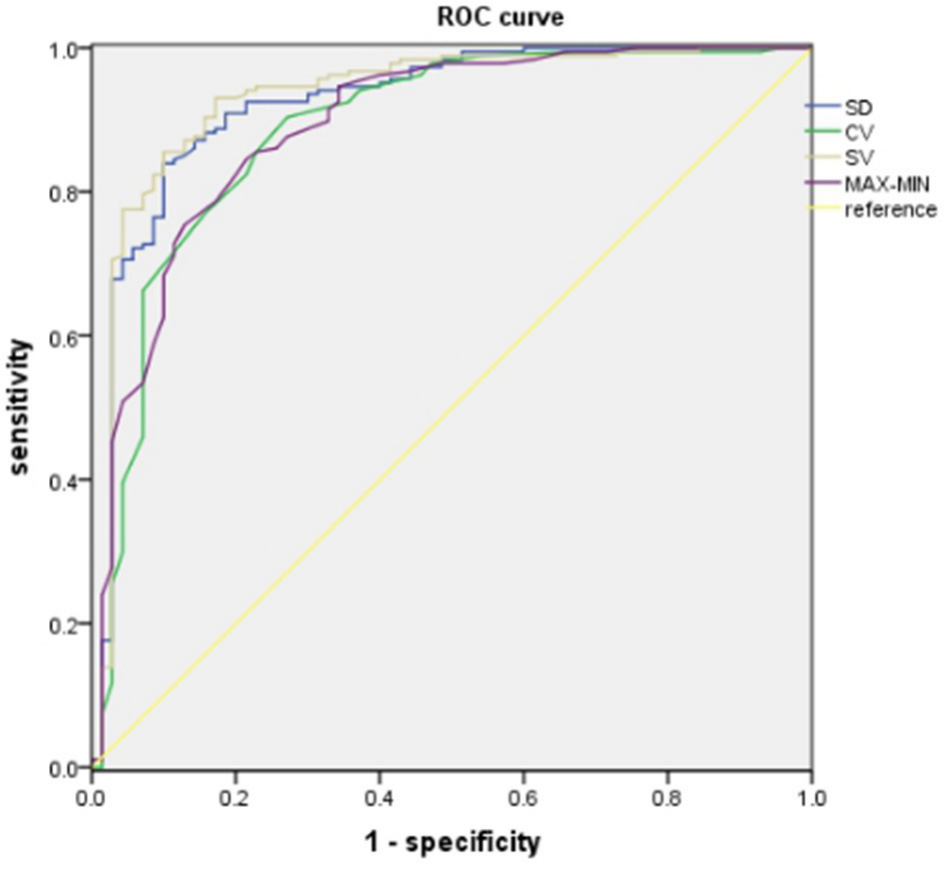
ROC analysis of PP variability and 3-month unfavorable outcomes. PP, pulse pressure; SD, standard deviation; CV, coefficient of variation; SV, successive variation; ΔBP, maximum–minimum difference.

The areas under the ROC curve for PPV parameters were 0.924 for SD, 0.886 for CV, 0.932 for SV, and 0.892 for ΔBP (*P* < 0.001; Figure 2). The optimal cutoff value (sensitivity, specificity, Youden Index) was 11.395 (0.840, 0.900, 0.740) for SD, 0.185 (0.904, 0.729, 0.633) for CV, 14.360 (0.930, 0.829, 0.759) for SV, and 16.545 (0.856, 0.900, 0.756) for ΔBP (Table 5).

**Table 5:** Cut-off values of PP variability.

**Values**	**AUC (95% CI)**	***P*-values**	**Best cut-off**	**Sensitivity (%)**	**Specificity (%)**	**Youden index**
SD	0.924 (0.882–0.965)	<0.001	11.395	0.840	0.900	0.740
CV	0.886 (0.835–0.938)	<0.001	0.185	0.904	0.729	0.633
SV	0.932 (0.891–0.973)	<0.001	14.360	0.930	0.829	0.759
ΔBP	0.892 (0.845–0.939)	<0.001	16.545	0.856	0.900	0.756

*PP, pulse pressure; AUC, areas under the curve; SD, standard deviation; CV, coefficient of variation; SV, successive variation; ΔBP, maximum–minimum difference*.

## Discussion

The clinical outcome of LVO patients treated with EVT is affected by many factors including age, hypertension, and stroke severity, among others (5, 19). This is supported by the results of the present study. We found that patients with poor outcomes were older and were more likely to have a history of hypertension, coronary artery disease, and valvular heart disease; stroke severity at admission was also worse in this group (Table 1). We also found that a high number of patients with successful recanalization had unfavorable outcomes, and more frequently patients treated with intravenous thrombolysis had unfavorable outcomes (Table 1). Although reperfusion and recanalization are closely related, these two events can evolve independently over time. Recanalization may fail to induce reperfusion because of severely damaged microcirculation (20). And successful recanalization of the occluded artery did not always translate into a good outcome for patients with acute LVO stroke (21, 22). The prognosis of successful recanalization patients may be related to many factors, such as stroke severity at admission, cerebral infarction volume, and symptom onset to recanalization time (23, 24). In the present study, the proportion of patients with poor outcomes was lower than the recent large thrombectomy trials (25, 26). It may be related to that the patients in this study with a greater stroke severity at admission, larger cerebral infarction volume, longer symptom onset to recanalization time, higher Glucose at admission, and so on.

Management of BP during mechanical thrombectomy is important because it is associated with functional outcome. There is increasing evidence that BPV is a strong predictor of prognosis in stroke patients (6, 16–19, 27, 28). In a study of 182 stroke patients treated with intra-arterial therapies (including mechanical thrombectomy, intra-arterial tissue plasminogen activator, intra-arterial abciximab, angioplasty, and/or stenting), multivariate regression analysis of the relationship between BPV and clinical outcome showed that increased BPV predicted a worse outcome (29). Decreased BPV in the first 24 h post-EVT was associated with favorable outcome at 3 months in LVO patients, and systolic SV of BPV was identified as a potential predictor of functional prognosis (5). The results of the current study (Tables 2, 4) are consistent with earlier reports that BPV is significantly associated with clinical outcome in patients treated with EVT (19, 29). PPV in the sICH group was higher than that in the non-sICH group (Table 3), suggesting that increased PPV enhances the risk of sICH in patients who have undergone IAT, and that PPV affects the clinical outcome of IAT patients. We also found that after adjusting for potential confounders, PPV was associated in a graded manner with poor outcome, the odds of which were significantly elevated in the highest tertile of PPV compared to the lowest tertile, indicating that greater variability corresponds to a stronger association (Table 4). The ROC curve analysis showed that all four PPV parameters had excellent predictive value for poor outcome at 3 months (Figure 2 and Table 5), demonstrating that PPV may be a useful predictor of functional prognosis in LVO patients treated with IAT.

There are several potential mechanisms linking BPV during the perioperative period of IAT with clinical outcome. Recurrent sudden rises and falls in BP may increase shear force in blood vessels and induce vascular inflammation through upregulation of endothelial cytokines, which could disrupt the blood–brain barrier and promote the formation of atherosclerotic plaques (30, 31). Increased BPV after AIS may enhance cell death in the area of impaired cerebral autoregulation as a result of greater fluctuation in cerebral blood flow (32), which depends on cerebral perfusion pressure and blood viscosity (33). A sudden drop in BP may reduce cerebral perfusion pressure and promote the expansion of the infarct core. On the other hand, a sudden increase in BP may increase the risk of cerebrovascular damage and cerebral hemorrhage. PPV reflects fluctuations in SBP and DBP; the present findings indicate that greater PPV during the perioperative period of IAT can lead to a worse outcome, possibly by amplifying secondary brain injury.

Our study had several limitations. First, although a recent study found that antihypertensive agents can affect BPV (34), we did not evaluate the relationship between BPV and the use of antihypertensive agents in this study. Second, the relationship between BPV and long-term outcome in LVO patients treated with EVT was not examined. Third, the management of LVO patients in this study—who were enrolled from October 2015 to January 2020—was not uniform, as they were treated according to the 2015 and 2018 Chinese guidelines for EVT of AIS. Fourth, the blood pressures that were recorded through cuff pressures may limit the validity of the results. Finally, the fact that this was a single-center retrospective study limits the generalizability of the results.

## Conclusions

The results of our study demonstrate that BPV from admission through the first 24 h after IAT is independently associated with poor outcome at 3 months in patients with LVO, with a greater variability corresponding to a stronger association. Thus, PPV may be a useful predictor of functional prognosis in LVO patients treated with IAT. Additionally, stabilization of BPV during the perioperative period of IAT may be an effective strategy to improve the outcome of LVO patients.

## Data Availability Statement

The raw data supporting the conclusions of this article will be made available by the authors, without undue reservation.

## Ethics Statement

The present study was approved by The Fourth Affiliated Hospital of Guangxi Medical University (No. LW2020009). Informed consent was obtained from the patients or their guardians.

## Author Contributions

MY and TL contributed to analyzed the data and drafted the manuscript. BW and HY contributed to the study design and interpreted the data. YH contributed to acquisition of data. All authors contributed to the article and approved the submitted version.

## Conflict of Interest

The authors declare that the research was conducted in the absence of any commercial or financial relationships that could be construed as a potential conflict of interest.
